# Deep Deconvolutional Neural Network for Target Segmentation of Nasopharyngeal Cancer in Planning Computed Tomography Images

**DOI:** 10.3389/fonc.2017.00315

**Published:** 2017-12-20

**Authors:** Kuo Men, Xinyuan Chen, Ye Zhang, Tao Zhang, Jianrong Dai, Junlin Yi, Yexiong Li

**Affiliations:** ^1^National Cancer Center/Cancer Hospital, Chinese Academy of Medical Sciences and Peking Union Medical College, Beijing, China

**Keywords:** automatic segmentation, target volume, deep learning, deep deconvolutional neural network, radiotherapy

## Abstract

**Background:**

Radiotherapy is one of the main treatment methods for nasopharyngeal carcinoma (NPC). It requires exact delineation of the nasopharynx gross tumor volume (GTVnx), the metastatic lymph node gross tumor volume (GTVnd), the clinical target volume (CTV), and organs at risk in the planning computed tomography images. However, this task is time-consuming and operator dependent. In the present study, we developed an end-to-end deep deconvolutional neural network (DDNN) for segmentation of these targets.

**Methods:**

The proposed DDNN is an end-to-end architecture enabling fast training and testing. It consists of two important components: an encoder network and a decoder network. The encoder network was used to extract the visual features of a medical image and the decoder network was used to recover the original resolution by deploying deconvolution. A total of 230 patients diagnosed with NPC stage I or stage II were included in this study. Data from 184 patients were chosen randomly as a training set to adjust the parameters of DDNN, and the remaining 46 patients were the test set to assess the performance of the model. The Dice similarity coefficient (DSC) was used to quantify the segmentation results of the GTVnx, GTVnd, and CTV. In addition, the performance of DDNN was compared with the VGG-16 model.

**Results:**

The proposed DDNN method outperformed the VGG-16 in all the segmentation. The mean DSC values of DDNN were 80.9% for GTVnx, 62.3% for the GTVnd, and 82.6% for CTV, whereas VGG-16 obtained 72.3, 33.7, and 73.7% for the DSC values, respectively.

**Conclusion:**

DDNN can be used to segment the GTVnx and CTV accurately. The accuracy for the GTVnd segmentation was relatively low due to the considerable differences in its shape, volume, and location among patients. The accuracy is expected to increase with more training data and combination of MR images. In conclusion, DDNN has the potential to improve the consistency of contouring and streamline radiotherapy workflows, but careful human review and a considerable amount of editing will be required.

## Introduction

Nasopharyngeal carcinoma (NPC) is a malignant tumor prevalent in southern China. Radiotherapy is one of the main treatments for NPC, and its rapid development has played a significant role in the improvement of tumor control probability. Intensity-modulated radiotherapy and volumetric-modulated radiotherapy (VMAT) have become the state-of-the-art methods for the treatment of NPC over the past two decades (1, 2). These technologies can facilitate dose escalation to the tumor target while improving the sparing of organs at risk (OARs), and the dose distribution usually has steep gradients at the target boundary. Modern treatment planning system (TPS) requires exact delineation of the nasopharynx gross tumor volume (GTVnx), the metastatic lymph node gross tumor volume (GTVnd), the clinical target volume (CTV) to be irradiated, and OARs to be spared in planning computed tomography (CT) images so that a radiation delivery plan can be optimized reversely. This task is a type of image segmentation and is usually carried out manually by radiation oncologists based on recommended guidelines (e.g., RTOG 0615 Protocol). However, the manual segmentation (MS) process is time-consuming and operator dependent. It has been reported that the segmentation of a single head-and-neck (H&N) cancer case takes an average of ~2.7 h (3). This time-consuming work may be repeated several times during a course of NPC radiotherapy due to a tumor response or significant anatomic changes and alterations. In addition, the accuracy of the segmentation is highly dependent on the knowledge, experience, and preference of the radiation oncologists. Considerable inter- and intra-observer variation in segmentation of these regions of interest (ROIs) have been noted in a number of studies (4–7).

As a result, a fully automated segmentation method for radiotherapy is helpful to relieve radiation oncologists from the labor-intensive aspects of their work and increase the accuracy, consistency, and reproducibility of ROI delineation. “Atlas-based segmentation” (ABS) (8–10) incorporates a prior knowledge into the process of segmentation and is one of the most widely used and successful image segmentation techniques for biomedical applications. In this type of method, an optimal transformation between the target image to be segmented and a single atlas or multiple atlases containing some ground truth segmentations is computed using deformable registration techniques. Then, all the labeled structures in the atlas image can be propagated through the registration transformation onto the target image automatically. ABS has become a popular method in automatic delineation of target and/or OARs in H&N radiotherapy (11–17) due to its acceptable results and fully unsupervised mode of operation. Han et al. (11) used the object shape information in the atlas to account for large inter-subject shape differences. Sjöberg et al. (12) applied fusion of multiple atlases to improve the segmentation accuracy than single atlas segmentation. Tao et al. (13) used ABS to reduce interobserver variation and improve dosimetric parameter consistency for OARs. Teguh et al. (14) evaluated autocontouring using ABS and found it was a useful tool for rapid delineation, although editing was inevitable. Sims et al. (15) did a pre-clinical assessment of ABS and showed that it exhibited satisfactory sensitivity; however, careful review and editing were required. Walker et al. (16) concluded ABS was timesaving in generating ROI in H&N, but attending physician approval remained vital. However, there are two main challenges using the ABS method. First, due to the anatomical variations of human organs, it is difficult to build a “universal atlas” for all human organs. The ROI may be considerably different according to the body shape and body size of the patient. The variability should be taken into account to construct a patient-specific atlas from all atlas images, but there are difficulties for target images with a large variability in shape and appearance. Second, a large disadvantage of using ABS is the large computation time that is involved in registering the target image to its atlas image (18). Moreover, it often requires the target image to be aligned to multiple atlases, which will increase the process of registration several times.

Deep learning methods have achieved enormous success in many computer vision tasks, such as image classification (19–21), object detection (22, 23), and semantic segmentation (24–26). Convolutional neural networks (CNNs) have become the most popular algorithm for deep learning (21, 27). CNNs consist of alternating convolutional and pooling layers to automatically extract multiple-level visual features and have made significant progress in computer-aided diagnosis and automated medical image analysis (28–31). Melendez et al. (29) applied multiple-instance learning for tuberculosis detection using chest X-rays and reported an AUC of 0.86. Hu et al. (30) proposed a liver segmentation framework based on CNNs and globally optimized surface evolution, yielding a mean Dice similarity coefficient (DSC) of 97%. Esteva et al. (31) trained a CNN using a large dataset to classify skin cancer and achieved higher accuracy than dermatologists. In addition, CNNs have been applied in the segmentation of many organs and substructures, such as cells (32), nuclei (33), blood vessels (34), neuronal structures (35), brain (36), ventricles (37), liver (38), kidneys (39), pancreas (40), prostate gland (41), bladder (42), colon (43), and vertebrae (44) with relatively better overlap compared with state-of-the-art methods. However, these studies have been confined mostly to the field of radiology.

Furthermore, there has been increasingly more interest in applying CNNs to radiation therapy (45–48). Recently, Ibragimov and Xing (49) used CNNs for OARs segmentation in H&N CT images and obtained DSC values that varied from 37.4% for chiasm to 89.5% for mandible. This was the first report on OAR delineation with CNNs in radiotherapy; however, no target was segmented. In this work, we developed a deep deconvolutional neural network (DDNN) for the segmentation of CTV, GTVnx, and GTVnd for radiotherapy of NPC. The experimental results show that the DDNN can be used to realize the segmentation of NPC targets while planning CT images. DDNN is an end-to-end architecture consisting of two important components, including an encoder and a decoder. Different from typical CNNs, we performed a reversed deconvolution at decoder networks to rebuild high-resolution feature maps from low-resolution ones. Our work is the first attempt at applying DDNN for the auto-segmentation of a target for the planning of radiotherapy in NPC.

## Materials and Methods

### Data Acquisition

A total of 230 patients diagnosed with NPC stage I or stage II that received radiotherapy during January 2011 to January 2017 in our department were included in our study. All patients were immobilized with a thermoplastic mask (head, neck, shoulder) in the supine position. Simulation contrast CT data were acquired on a Somatom Definition AS 40 (Siemens Healthcare, Forchheim, Germany) or Brilliance CT Big Bore (Philips Healthcare, Best, the Netherlands) system set on helical scan mode with contrast enhancement. CT images were reconstructed using a matrix size of 512 × 512 and thickness of 3.0 mm. MR images of all patients were acquired to assist the definition of the targets. Radiation oncologists contoured the GTVnx, the GTVnd, CTV, and OARs in the planning CT using a Pinnacle TPS (Philips Radiation Oncology Systems, Fitchburg, WI, USA) system. The GTVnx was defined as the primary nasopharyngeal tumor mass. The GTVnd was defined as the metastatic lymph nodes. The CTV (CTV1 + CTV2) included GTVnx, GTVnd, high-risk local regions that contain the parapharyngeal spaces, the posterior third of nasal cavities and maxillary sinuses, pterygoid processes, pterygopalatine fossa, the posterior half of the ethmoid sinus, cavernous sinus, base of skull, sphenoid sinus, the anterior half of the clivus, petrous tips, and high-risk lymphatic drainage areas, including bilateral retropharyngeal lymph nodes and level II.

### DDNN Model for Segmentation

**Figure 1:**
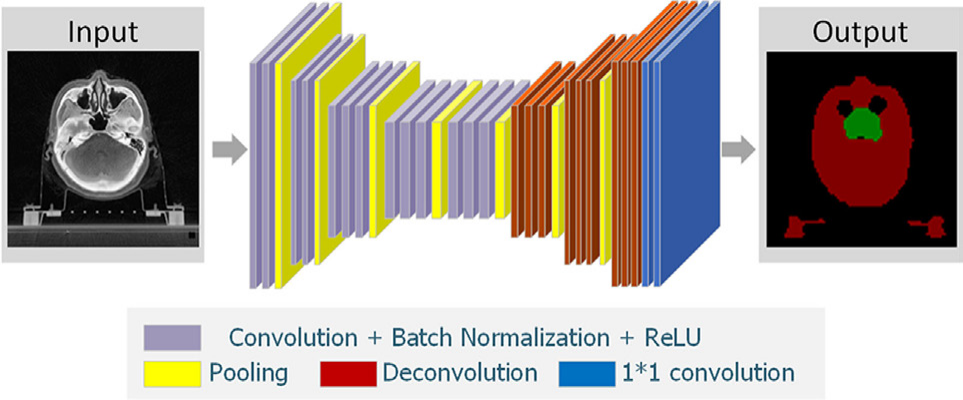
Overall framework of the proposed algorithm.

**Figure 2:**
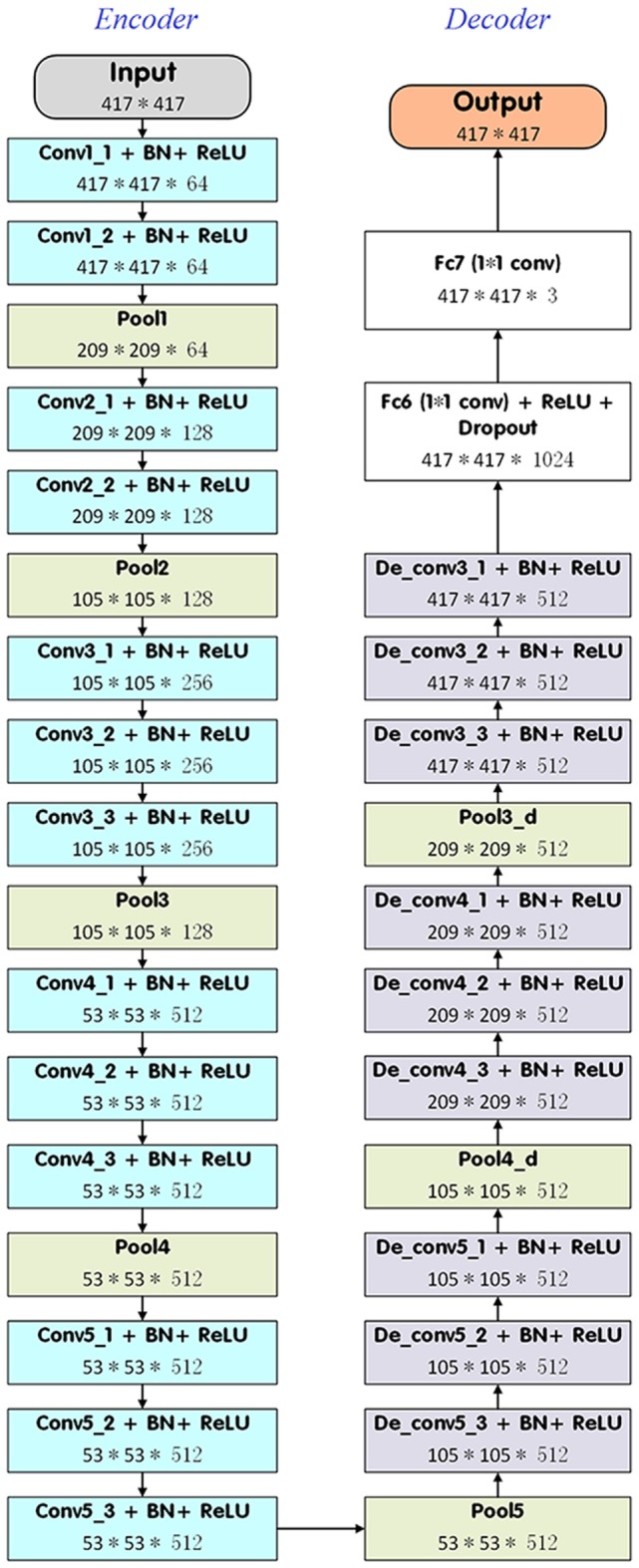
The detailed architecture of deep deconvolutional neural network.

In the present study, we introduced a DDNN model to segment the target NPC for radiotherapy. DDNN is an end-to-end segmentation framework that can predict pixel class labels in CT images. Figure 1 depicts the flowchart of the proposed model. As is shown in Figure 2, the DDNN networks consisted of two important components, including an encoder part and a decoder part. The encoder network consisted of 13 convolutional layers for feature extraction and was used to extract the visual features of the medical image, and the decoder network recovered the original resolution by deploying deconvolution. Specifically, the encoder network layers were based on the VGG-16 architecture (21), used for high-quality image classification. Different from VGG-16, we performed a reversed deconvolution at decoder networks to rebuild high-resolution feature maps from low-resolution. In addition, we replaced the fully connected layers with fully convolutional layers for our segmentation task. With the adaptation, the networks can achieve pixel segmentation in CT images. Please refer to the appendix for more technical specifications of the architecture.

### Experiments

Data from 184 patients out of 230 were chosen randomly as a training set to adjust the parameters of the DDNN model, and the remaining 46 patients were used as the test set to evaluate the performance of the model. In this work, we implemented our model’s training, evaluation, error analysis, and visualization pipeline using Caffe (50), which is a popular deep learning framework, and then compiled using cuDNN (51) computational kernels. For the experiments, we adopted data augmentation techniques, such as random cropping and flipping to reduce over fitting. We used stochastic gradient descent with momentum to optimize the loss of function. We set the initial learning rate to 0.0001, learning rate decay factor to 0.0005, and decay step size to 2,000. Instead of using a fixed number of steps, we trained our model until the mean average precision of the training set converged, and then evaluated the model using the validation set. We used NVIDIA TITAN XP GPU for all experiments.

### Quantitative Evaluation

A total of 46 patients were used to assess the performance of the model. MSs were defined as the reference segmentations generated by the experienced radiation oncologists. All the voxels that belong to the MS were extracted and labeled. During the testing phase, all the 2D CT slices were tested one by one. The input was the 2D CT image, and the final output was pixel-level classification, which was the most likely classification label. Performance of the proposed method was tested and compared with the segmentation of the GTVnx, GTVnd, and CTV. The DSC and the Hausdorff distance (*H*) were used to quantify the results.

The DSC is defined as shown in Eq. 1 as follows:

(1) $$\text{DSC}(\text{A},\text{B})=\frac{2|\text{A}\cap \text{B}|}{\left|\text{A}|+|\text{B}\right|}$$

where A represents the MS, B denotes the auto-segmented structure and A ∩ B is the intersection of A and B. The DSC results in values between 0 and 1, where 0 represents no intersection at all and 1 reflects perfect overlap of structures A and B.

The Hausdorff distance (*H*) is defined as

(2) H(A,B)=max(h(A,B),h(B,A))

where

(3) $$h(\text{A},\text{B})=\underset{a\in A,b\in B}{\text{max}(\text{min}\|a-b\|)}$$

and ‖.‖ is some underlying norm on the points of A and B. As *H*(A,B) diminishes, the overlap between A and B increases.

In addition, the performance of DDNN was compared with VGG-16. The average DSC and Hausdorff distance values for the three targets (GTVnx, GTVnd, and CTV) were analyzed with paired *t*-tests between DDNN and VGG-16. All analyses were performed with a *p*-value set to <0.05.

## Results

**Figure 3:**
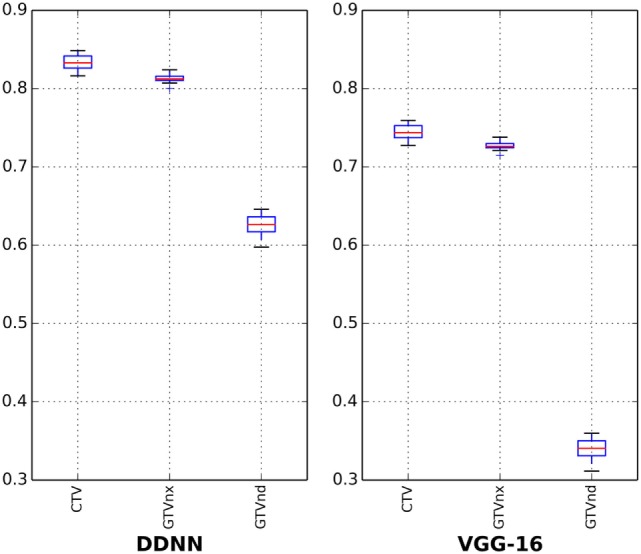
Boxplots obtained from Dice similarity coefficient analyses.

The results for all tested patients and GTVnx, GTVnd, and CTV values are summarized in Figure 3 and Table 1. The proposed DDNN auto-segmentation showed a better overall agreement than the VGG-16 based auto-segmentation, as shown by the DSC values. The average DSC value of DDNN was 15.4% higher than the VGG-16 average DSC value (75.3 ± 11.3 vs. 59.9 ± 22.7%, *p* < 0.05). Automatic delineation with DDNN produced a good result for the GTVnx and CTV, with DSC values of 80.9 and 82.6%, respectively. These values showed a reasonable volume overlap of the auto-segmented contours and the manual contours. The quality of the automatically generated GTVnd was barely satisfactory, with a mean DSC value of 62.3%. The Hausdorff distance values for all targets were reduced by DDNN compared with VGG-16 (12.6 ± 11.5 vs. 23.4 ± 24.4, *p* < 0.05).

**Table 1:** Dice similarity coefficient (DSC) and Hausdorff distance for nasopharynx gross tumor volume (GTVnx), metastatic lymph node gross tumor volume (GTVnd), and clinical target volume (CTV).

	DSC (%)			Hausdorff distance (mm)		
Region of interest	CTV	GTVnx	GTVnd	CTV	GTVnx	GTVnd
Deep deconvolutional neural network	82.6	80.9	62.3	6.9	5.1	25.8
VGG-16	73.7	72.3	33.7	11.1	7.7	51.5

**Figure 4:**
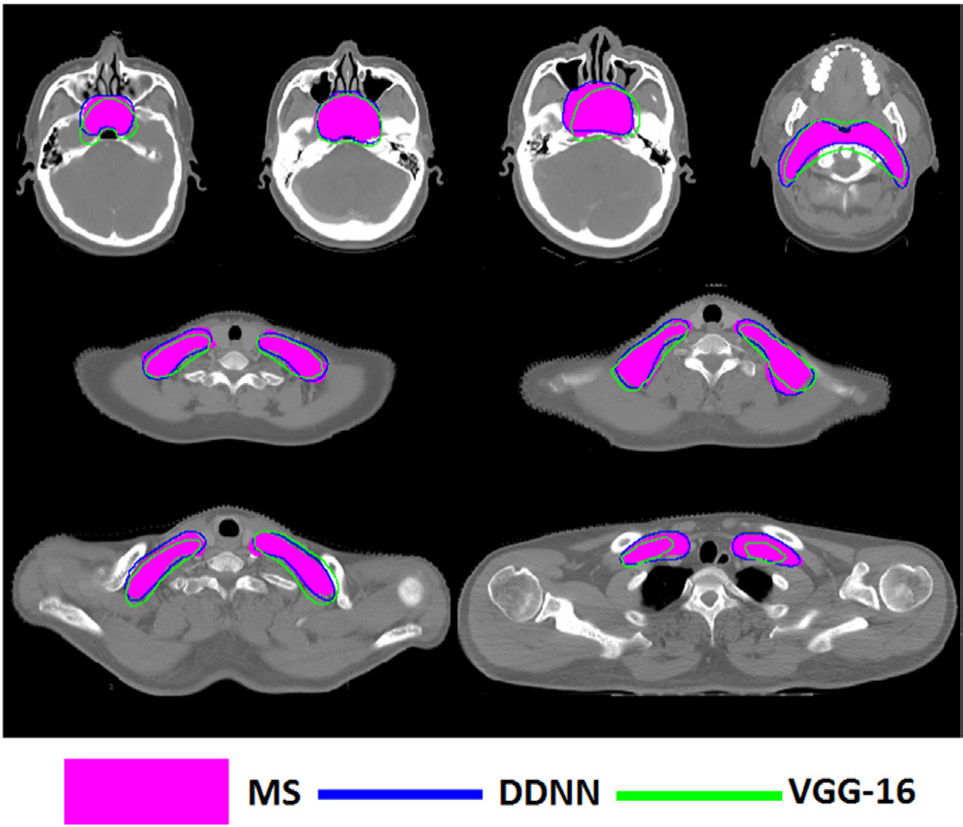
Segmentation results for clinical target volume, shown in transverse computed tomography slices.

**Figure 5:**
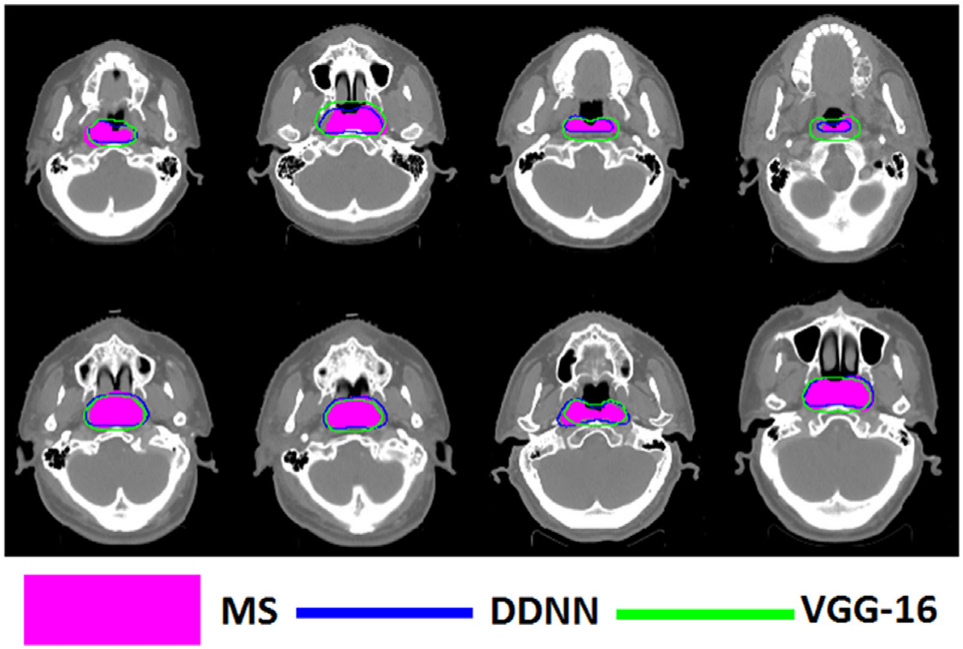
Segmentation results for nasopharynx gross tumor volume, shown in transverse computed tomography slices.

**Figure 6:**
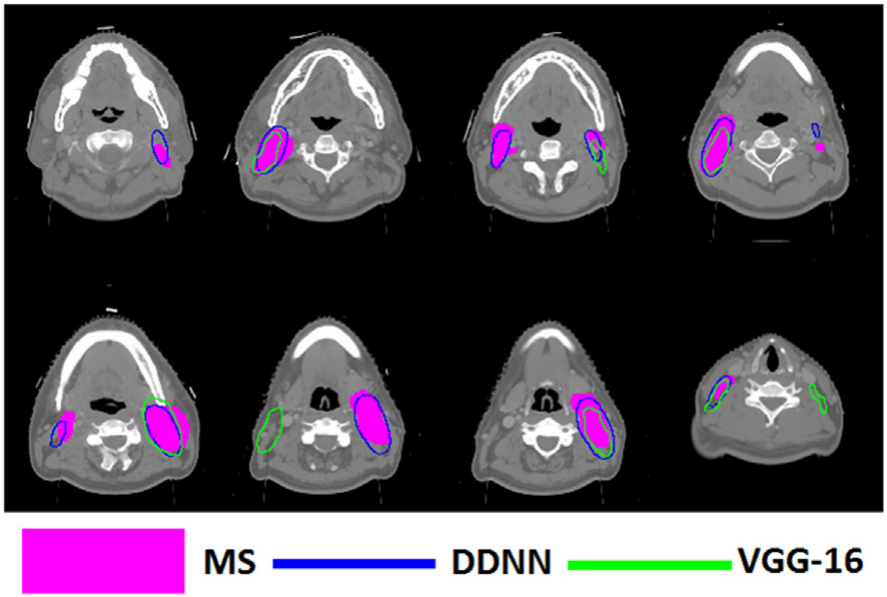
Segmentation results for metastatic lymph node gross tumor volume, shown in transverse computed tomography slices.

Figures 4–6 show auto-segmentation of CTV, GTVnx, and GTVnd for test cases, respectively. In these examples, the auto-segmented contours of CTV and GTVnx using DDNN were close to the MS contours, although inconsistencies existed. Only a few corrections were necessary to validate the automatic segmentation. However, for the segmentation of the GTVnd, there was some deviation from the MS in shape, volume, and location.

## Discussion

We have designed an automated method to segment CT images of NPC. To the best of our knowledge, this task has not previously been reported. Our results suggest that the proposed DDNN algorithm can learn the semantic information from nasopharyngeal CT data and produce high-quality segmentation of the target. We compared the proposed architecture with the popular Deeplab v2 VGG-16 model. This comparison revealed that our method achieved better segmentation performance. Our DDNN method deployed a deeper encoder and decoder neural network, which used convolutional filters to extract feature and deployed deconvolutional filters to recover the original resolution. Thus, detailed segmented results were learned/predicted better than bilinear interpolation.

Consistency of target delineation is essential for the improvement of radiotherapy outcomes. Leunens et al. (52) demonstrated that inter- and intra-observer variation is considerable. Lu et al. (53) investigated the interobserver variations in GTV contouring of H&N patients and reported a DSC value of only 75%. Caravatta et al. (54) evaluated the overlap accuracy of CTV delineation among different radiation oncologists and got a DSC of 68%. Automatic segmentation has the potential to reduce variability of contours among physicians and improve efficiency. The gains in efficiency and consistency are valuable only if accuracy is not compromised. Assessment of accuracy of a segmentation method is complex, because there is no common database or objective volume for comparison. The evaluation of automatic segmentation for radiotherapy planning usually uses the DSC value, thus providing a reasonable basis for comparison. Apparently, our method showed good performance compared with the existing studies regarding the auto-segmentation topic. In addition, such auto-segmentation methods are atlas- and/or model based, and there is no report on segmentation of GTV or CTV using a deep learning method. Regarding the target, the comparison is difficult since *N*-stage (most often *N*0) and selected levels were quite different from one study to another. For CTV, different previous publications reported mean DSC values of 60% (55), 60% (8), 60% (56), 67% (14), 77% (57), 78% (58), 79% (59), and 80.2% (60), whereas the DSC value of DDNN was 82.6%. There are few reports on auto-segmentation of GTVnx or GTVnd. For segmentation of GTVnx, DSC values have been reported to be 69.0% (58) and 75.0% (61), whereas our proposed method demonstrated a high DSC value of 80.9%. The segmentation of GTVnd reported in the literature has yielded DSC values of 46.0% (62), and our method showed a DSC value of 62.3%. It is unfair to say our proposed algorithm is superior because the comparison with the published methods was not done with the same dataset; however, it is reasonable to conclude DDNN resulted in good results. Meanwhile, the proposed method learns and predicts in an end-to-end form without post-processing, which makes the inference time of the whole network within seconds.

Although the segmentation accuracy for GTVnd was better than previously reported, it was still too low. There are several reasons for this deficiency. First, this low result was due to lack of soft tissue contrast in CT-based delineation. Second, the GTVnd typically does not have constant image intensity or clear anatomic boundaries, and its shape and location are more variable compared with CTV and GTVnx among different patients. Moreover, there is no GTVnd region in *N*0 patients, who were also included in our training and test sets. All of these factors will hinder the DDNN model from learning the robust features and making accurate reasoning. Thus, the segmentation accuracy of GTVnd remains unsatisfactory at present. Zijdenbos et al. (63) suggests that a DSC value of >70% represents good overlap. Although the segmentation accuracy of CTV and GTVnx exceeded this standard, attending physician oversight remains critical. Imperfect definition of target volumes, which are then used for treatment planning, may result in under dosage of target volumes or an overdose delivered to normal tissues. As a result, the proposed method cannot be applied in an unsupervised fashion in the clinic. Human review and a considerable amount of editing might be required.

There are several limitations to our study. First, a model trained on *N*0 and *N*+ patients was used to assess the testing set, including both *N*0 and *N*+ patients. This may make the model difficult to converge and reduce the accuracy of the prediction. Second, only one physician delineated the target for each patient but all the patients were delineated by several observers. Although the targets were contoured by experts according to the same guideline for NPC, there was still interobserver variability in all cases. We cannot exclude such possible bias, which challenges the DDNN method. Another limitation of our study is that all of the included patients were stage I or stage II. A target with different stages may have different contrast, shapes, and volumes, thus, influencing the performance of the automated segmentation.

This study mainly focused on NPC target segmentation from CT images. However, MR images in H&N have superior soft-tissue contrast and the GTV delineation often depends on MR images. In addition, functional MR may allow accurate location of the tumors. In the future, DDNN is expected to combine with the MR or other types of images to improve target volume delineation. The training set included only 184 patients. Increasing the amount of training data could make the DDNN model more robust, improving the segmentation accuracy. With the initiation of improved target visualization and further improvement of segmentation algorithms in the future, accuracy of auto-segmentation is likely to improve.

## Conclusion

Accurate and consistent delineation of tumor target and OARs is particularly important in radiotherapy. Several studies have focused on the segmentation of OARs using deep learning methods. This study shows a method using DDNN architecture to auto-segment nasopharyngeal cancer stage I or stage II in planning CT images. The results suggest that DDNN can be used to segment GTVnx and CTV with high accuracy. The accuracy for GTVnd segmentation was relatively low due to the considerable differences in shape, volume, and location among patients. The performance is expected to improve with multimodality medical images and more training data. In conclusion, DDNN has the potential to improve the consistency of contouring and streamline radiotherapy workflows, but careful human review and a considerable amount of editing will be required.

## Availability of Data and Materials

The datasets generated and/or analyzed during the current study are not publicly available due to data security but are available from the corresponding author on reasonable request.

## Ethics Statement

This study was carried out in accordance with the Declaration of Helsinki and was approved by the Independent Ethics Committee of Cancer Hospital, Chinese Academy of Medical Sciences with the following reference number: NCC2015 YQ-15.

## Author Contributions

All authors discussed and conceived of the study design. KM wrote the programs and performed data analysis, and drafted the manuscript. XC and YZ analyzed and interpreted the patients’ data. JD, JY, and YL guided the study and participated in discussions and preparation of the manuscript. All authors read, discussed, and approved the final manuscript.

## Conflict of Interest Statement

The authors declare that the research was conducted in the absence of any commercial or financial relationships that could be construed as a potential conflict of interest.

## Abbreviations

NPC, nasopharyngeal carcinoma; GTVnx, nasopharynx gross tumor volume; GTVnd, metastatic lymph node gross tumor volume; CTV, clinical target volume; OARs, organs at risk; CT, computed tomography; H&N, head and neck; GT, ground truth; CNNs, convolutional neural networks; DDNN, deep deconvolutional neural network; DSC, Dice similarity coefficient; TCP, tumor control probability; IMRT, intensity-modulated radiotherapy; VMAT, volumetric-modulated radiotherapy; TPS, treatment planning system; ROIs, regions of interest; ABS, Atlas-based segmentation; BN, batch normalized; ReLU, rectified linear non-linearity.

## Appendix

### Architecture of Deep Deconvolutional Neural Network (DDNN)

As is shown in Figure 2, the architecture of the proposed DDNN consisted of two parts, each of which had its own role. The encoder networks consisted of 13 convolutional layers for feature extraction. All the kernels of convolutional layers had a window size of 3 × 3, a stride of 1, and a padding of 1 pixel. In addition, there was a batch normalized option following each convolution layer and then an element-wise rectified linear non-linearity max (0, *x*) was applied. The pooling options were added after the layers of conv1_2, conv2_2, conv3_3, conv4_3, conv5_3, de_conv5_1, and de_conv4_1 in order to get the robust feature. Specifically, the input size of the medical images in this work was cropped to 417 × 417 with 3 channels. Conv1_1 and conv1_2 convolved the input to 417 × 417 × 64, and then reduced to 209 × 209 × 64 feature maps using pooling option with kernel size of 3 × 3, a stride of 2, and a padding of 1 pixel. Similarly, the layers of conv2_1 and conv2_2 took pool1 as input. After using 3 × 3 convolution with a stride of 1 and a padding of 1 pixel, it produced 105 × 105 × 256 feature maps and then was pooled by pool2 and convolved by conv3, conv4, and conv5. The max pooling options of pool4 and pool5 with 3 × 3 filter size, pad 1 and stride 1, resulted in a 53 × 53 × 512 output. The pooling options reduced the spatial size of feature map, so the feature map needed to be recovered to the original spatial size for segmentation task. Most previous methods used bilinear interpolation to get high-resolution image; however, a coarse segmentation was not enough to produce good performance for nasopharyngeal cancer. Therefore, the decoder part deployed a deep deconvolution neural network which took pool5 as input and a serial of deconvolution layers for upsampling. All the deconvolutional layers used 3 × 3 convolution with the padding size of 1. At de_conv5_3, de_conv4_3, and de_conv3_3, the stride was set to 2. For others, the stride was set to 1. After 8× enlarging, the feature maps recovered the high resolution as same as input. At fc6 and fc7 layers, we replaced fully connected layer with 1 × 1 convolution. Thus, we can carry on the pixel-level classification for the segmentation task. The final outputs generated predicted label for each pixel.
